# Roflumilast Inhibits Fibrogenic Activation in Human Intestinal Myofibroblasts via Inhibition of Myocardin-Related Transcription Factor/Serum Response Factor Signaling

**DOI:** 10.3390/ijms27167126

**Published:** 2026-08-08

**Authors:** Dongju Lee, Yoon Jeong Choi, In Kyung Yoo, Jeonghee Han, Duk Hwan Kim, Jee Hyun Kim, Jun Hwan Yoo

**Affiliations:** 1 Department of Gastroenterology, CHA Bundang Medical Center, CHA University School of Medicine, Seongnam 13496, Republic of Korea; dongju9429@naver.com (D.L.); jeje3127@naver.com (Y.J.C.); ikyoo82@hanmail.net (I.K.Y.); teires.d.kim@gmail.com (D.H.K.); 2 Institute of Basic Medical Sciences, CHA University School of Medicine, Seongnam 13496, Republic of Korea; 3 Department of Surgery, CHA Bundang Medical Center, CHA University School of Medicine, Seongnam 13496, Republic of Korea; drhjh81@cha.ac.kr; 4 Division of Gastroenterology, Department of Internal Medicine, Seoul Metropolitan Government—Seoul National University Boramae Medical Center, Seoul National University College of Medicine, Seoul 07061, Republic of Korea

**Keywords:** roflumilast, Phosphodiesterase 4 inhibitors, human intestinal myofibroblasts, myocardin-related transcription factor, serum response factor

## Abstract

The lack of effective anti-fibrotic agents remains a significant unmet need in the treatment of intestinal fibrosis, a condition largely driven by transforming growth factor-β1 (TGF-β1)-mediated myofibroblast activation. Phosphodiesterase 4 (PDE4) inhibitors, known to increase intracellular cAMP levels, exhibit anti-inflammatory and potential anti-fibrotic properties, though their efficacy in intestinal fibrosis is poorly defined. In this study, we investigated the anti-fibrotic effects and underlying mechanisms of PDE4 inhibitors (rolipram, roflumilast, piclamilast, and mesopram) in human intestinal myofibroblasts (HIMFs) stimulated with TGF-β1. All PDE4 inhibitors significantly reduced TGF-β1-induced mRNA expression of collagen1A1 (*COL1A1*) and fibronectin (*FN1*), with mesopram additionally reducing α-smooth muscle actin (*ACTA2*) mRNA expression. At the protein level, however, only roflumilast significantly decreased procollagen1A1 (Procol1A1) and FN expression. Mechanistically, roflumilast, rolipram, and mesopram significantly reduced serum response factor (*SRF*) mRNA expression, whereas piclamilast and mesopram significantly inhibited myocardin-related transcription factor A (*MRTFA*) mRNA expression. Rolipram and roflumilast exhibited a decreasing trend in *MRTFA* mRNA expression without statistical significance. Notably, roflumilast demonstrated superior inhibitory effects, significantly decreasing both MRTF-A and SRF protein expression and effectively reducing nuclear localization of MRTF-A. Additionally, rolipram and roflumilast significantly attenuated TGF-β1-induced phosphorylation of Smad2. These findings indicate that PDE4 inhibitors, particularly roflumilast, effectively suppress TGF-β1-induced fibrogenic activation in HIMFs by modulating MRTF/SRF and Smad-dependent pathways, highlighting the value of roflumilast for further preclinical investigation as a potential therapeutic strategy for intestinal fibrosis.

## 1. Introduction

Intestinal fibrosis, commonly associated with chronic inflammation in inflammatory bowel disease, is characterized by excessive collagen deposition, potentially resulting in intestinal failure. Despite the efficacy of existing anti-inflammatory treatments, they have limited impact on fibrosis [1,2]. Notably, intestinal fibrosis can persist and worsen independently of ongoing inflammation [3]. The lack of effective anti-fibrotic drugs and the self-propagating nature of fibrosis highlight the urgent need for novel approaches to anti-fibrotic therapeutics [4].

Intestinal fibrosis involves the intricate overproduction and accumulation of extracellular matrix (ECM) proteins, primarily collagen and fibronectin (FN). Activated myofibroblasts expressing α-smooth muscle actin (α-SMA) are central contributors to this excessive ECM deposition. These myofibroblasts are driven by the pro-fibrotic cytokine transforming growth factor-beta (TGF-β), mediated through a serum response factor (SRF)-dependent gene transcription program [4,5,6]. The signaling axis involving Ras homolog family member A (RhoA), Rho-associated protein kinase (ROCK), actin, myocardin-related transcription factor (MRTF), and SRF is critical in this process, affecting cell adhesion, migration, and cytoskeletal remodeling [7,8,9,10,11]. Dysregulation of this pathway has been linked to fibrosis in various organs [12,13,14,15,16].

HIMFs possess unique adaptations to the gastrointestinal tract, making them essential for maintaining gut homeostasis, mucosal barrier function, and immunological responses. Their continuous exposure to diverse antigens, mechanical stress from peristaltic movements, and rapid cellular turnover necessitate specialized functions and ECM composition distinct from fibroblasts in other organs. Their dynamic responses to cytokines like TGF-β1 result in differentiation into myofibroblasts, driving intestinal fibrosis through excessive ECM production. Furthermore, their close interactions with epithelial and immune cells are integral to gut mucosal immunity and tissue repair processes. These distinctive attributes emphasize the critical need for detailed study of HIMFs in their native microenvironment to better understand their specific roles in fibrosis and potential therapeutic targets [17,18,19,20].

Phosphodiesterases (PDEs), particularly the PDE4 family, play a crucial role in regulating cyclic adenosine monophosphate (cAMP), a key messenger in various cellular processes [17]. PDE4 inhibitors, which increase cAMP levels, have broad anti-inflammatory effects, making them significant therapeutic targets for various inflammatory disorders, including psoriasis, asthma, and COPD [18,19,20,21,22]. In addition to its anti-inflammatory effects, the anti-fibrotic effects and mechanisms of PDE4 inhibitors have been studied in several organs, such as the lung, skin, kidney, and liver [23,24,25]. Nonetheless, data on the anti-fibrotic effects of PDE4 inhibitors on intestinal fibrosis is limited. Elevated cAMP levels exert anti-fibrogenic effects through multiple mechanisms. One well-documented pathway involves the activation of protein kinase A (PKA) and cAMP response element-binding protein (CREB) signaling, which suppresses TGF-β-induced fibrogenic gene transcription. In addition, cAMP also activates Epac, which inhibits Smad2 phosphorylation through interactions with TGF-β receptor type I (TGFβR1/ALK5), highlighting a CREB-independent mechanism [26,27]. In experimental chronic colitis models, rolipram and apremilast have demonstrated efficacy in reducing collagen deposition and modulating the cAMP/PKA/CREB pathway, resulting in improved clinical symptoms and reduced tissue fibrosis [28,29].

This study aims to investigate the potential of PDE4 inhibitors (rolipram, roflumilast, piclamilast, and mesopram) to inhibit the fibrogenic activation of HIMFs and to unravel the underlying intracellular mechanisms responsible for their inhibitory effects, offering insights into their applicability as therapeutic interventions in intestinal fibrosis.

## 2. Results

### 2.1. PDE4 Inhibitors’ Impact on Cell Viability in Human Intestinal Myofibroblasts

**Figure 1:**
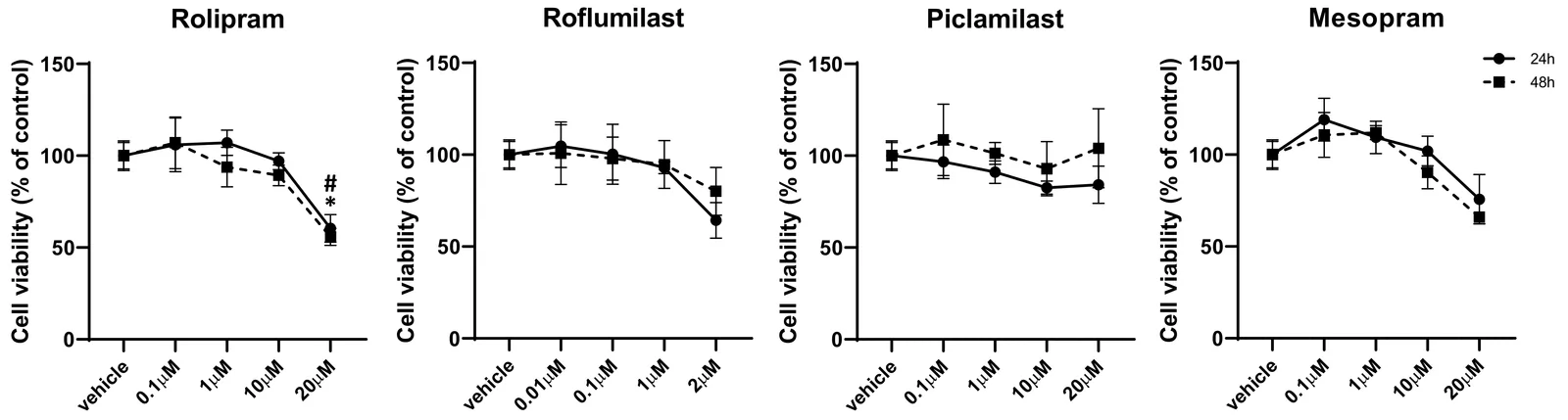
PDE4 inhibitors’ impact on cell viability in HIMF. HIMFs were treated with indicated concentration of rolipram, roflumilast, piclamilast or mesopram for 24 or 48 h, following which the cell viability was measured with the MTT assay (*n* = 3). Data are expressed as the means ± SEM. *^#^* *p* < 0.05 and * p < 0.05 versus the vehicle (24 or 48 h) (ANOVA w/Tukey).

To eliminate the possibility that the observed effects of PDE4 inhibitors resulted from cytotoxicity, we conducted a cell viability assay following the administration of PDE4 inhibitors at varying concentrations: rolipram (0.1–10 μM), roflumilast (0.01–2 μM), piclamilast (0.1–20 μM), or mesopram (0.1–20 μM) for either 24 or 48 h. Remarkably, none of the tested PDE4 inhibitors demonstrated any significant detrimental impact on cell viability at any concentration, with the exception of 20 μM of rolipram (Figure 1). Statistical analysis confirmed that there were no noteworthy differences in cell viability between the control group and the treatment groups across all concentrations of the PDE4 inhibitors tested.

### 2.2. Inhibition of TGF-β1-Induced ECM and α-SMA Expression by PDE4 Inhibitors in Human Intestinal Myofibroblasts

**Figure 2:**
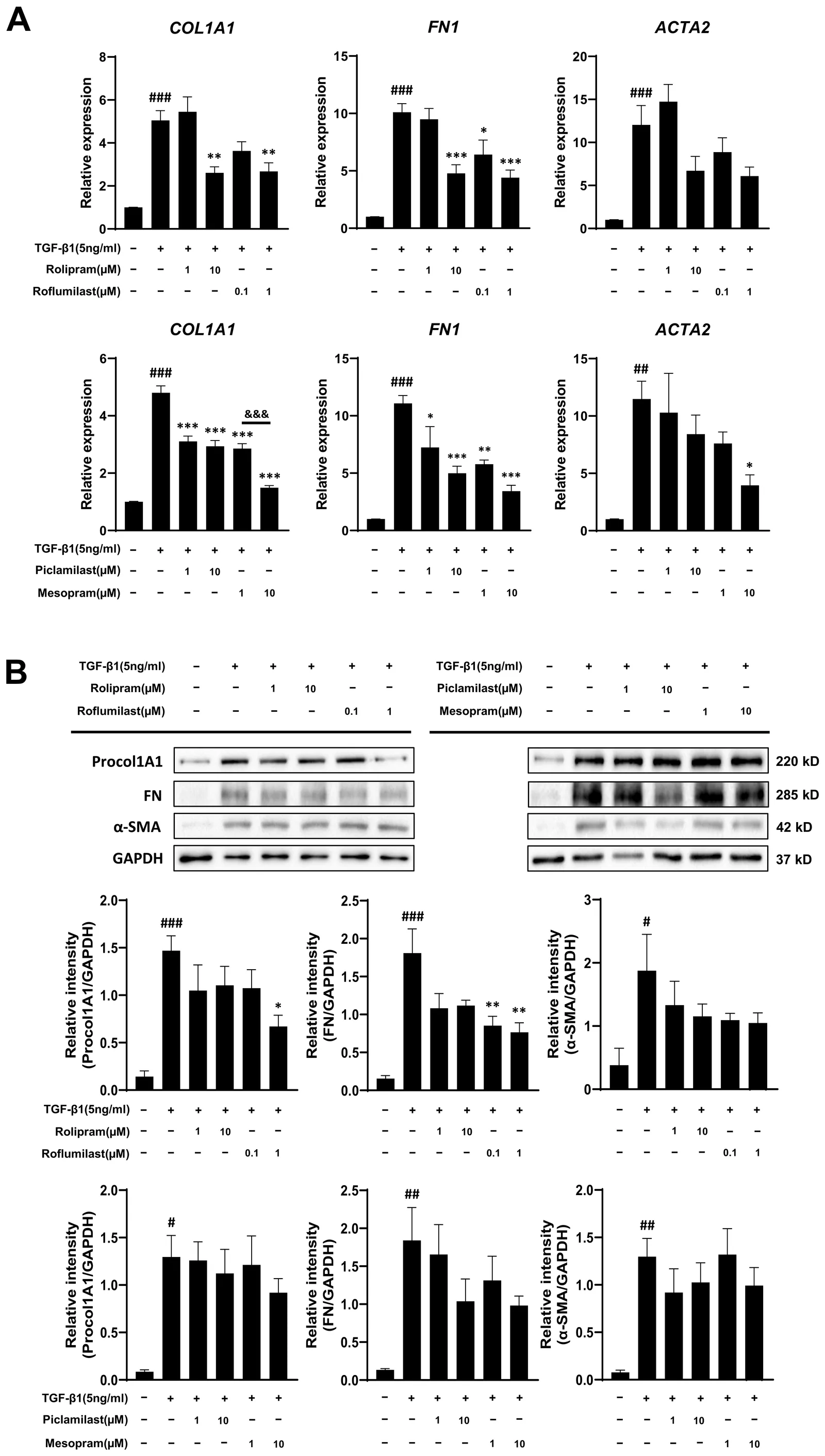
Co-culture with PDE4 inhibitors inhibits TGF-β1-induced fibrogenic activation of HIMF. HIMFs were treated with TGF-β1 (5 ng/mL) and co-cultured with or without PDE4 inhibitors (24 h for RT-qPCR analysis, 48 h for Western blotting). (**A**) RT-qPCR analysis of the relative mRNA expression of collagen1A1 (*COL1A1*), FN (*FN1*), and α-smooth muscle actin (*ACTA2*). The data were normalized to *GAPDH* expression and expressed as relative values compared to the control (rolipram, *n* = 7; roflumilast, *n* = 7; piclamilast, *n* = 4; and mesopram, *n* = 4). (**B**) Representative Western blots show the protein expression of procollagen1A1 (Procol1A1), fibronectin (FN), and α-smooth muscle actin (α-SMA), with GAPDH as a loading control. Quantitation of Procol1A1, FN, and α-SMA from Western blot analyses (rolipram, *n* = 6; roflumilast *n*, = 6; piclamilast, *n* = 7; and mesopram, *n* = 7). Data are expressed as the means ± SEM. ^#^ *p* < 0.05, ^##^ *p* < 0.01 and ^###^ *p* < 0.001 versus the control; * *p* < 0.05, ** *p* < 0.01 and *** *p* < 0.001 versus the TGF-β1 treatment only (ANOVA w/Tukey); ^&&&^ *p* < 0.001 compared 1 μM of mesopram group (Mann–Whitney U test).

To assess the potential of PDE4 inhibitors to hinder the fibrogenic activation of myofibroblasts, we co-cultured HIMFs with PDE4 inhibitors (rolipram, roflumilast, piclamilast, or mesopram) and then stimulated them with TGF-β1. Stimulation with TGF-β1 significantly upregulated the mRNA expression levels of collagen1A1 (*COL1A1*), FN (*FN1*), and α-SMA (*ACTA2*) in the HIMFs (Figure 2A). Co-culturing with rolipram (10 µM), roflumilast (0.1 µM or 1 µM), or piclamilast (1 µM or 10 µM) inhibited *COL1A1* and *FN1* expression while showing a decreasing trend in *ACTA2* expression. Additionally, mesopram (1 µM or 10 µM) significantly inhibited the expression of all three markers. These anti-fibrotic effects of PDE4 inhibitors were also observed at the protein level, where TGF-β1 induced an increase in protein expression of Procol1A1, FN, and α-SMA (Figure 2B and Figure S1). Roflumilast significantly reduced expression of Procol1A1 and FN; rolipram, piclamilast, and mesopram induced decreasing trends without statistical significance. Rolipram and roflumilast decreased α-SMA protein expression without statistical significance, whereas piclamilast and mesopram had no significant effect on α-SMA.

**Figure 3:**
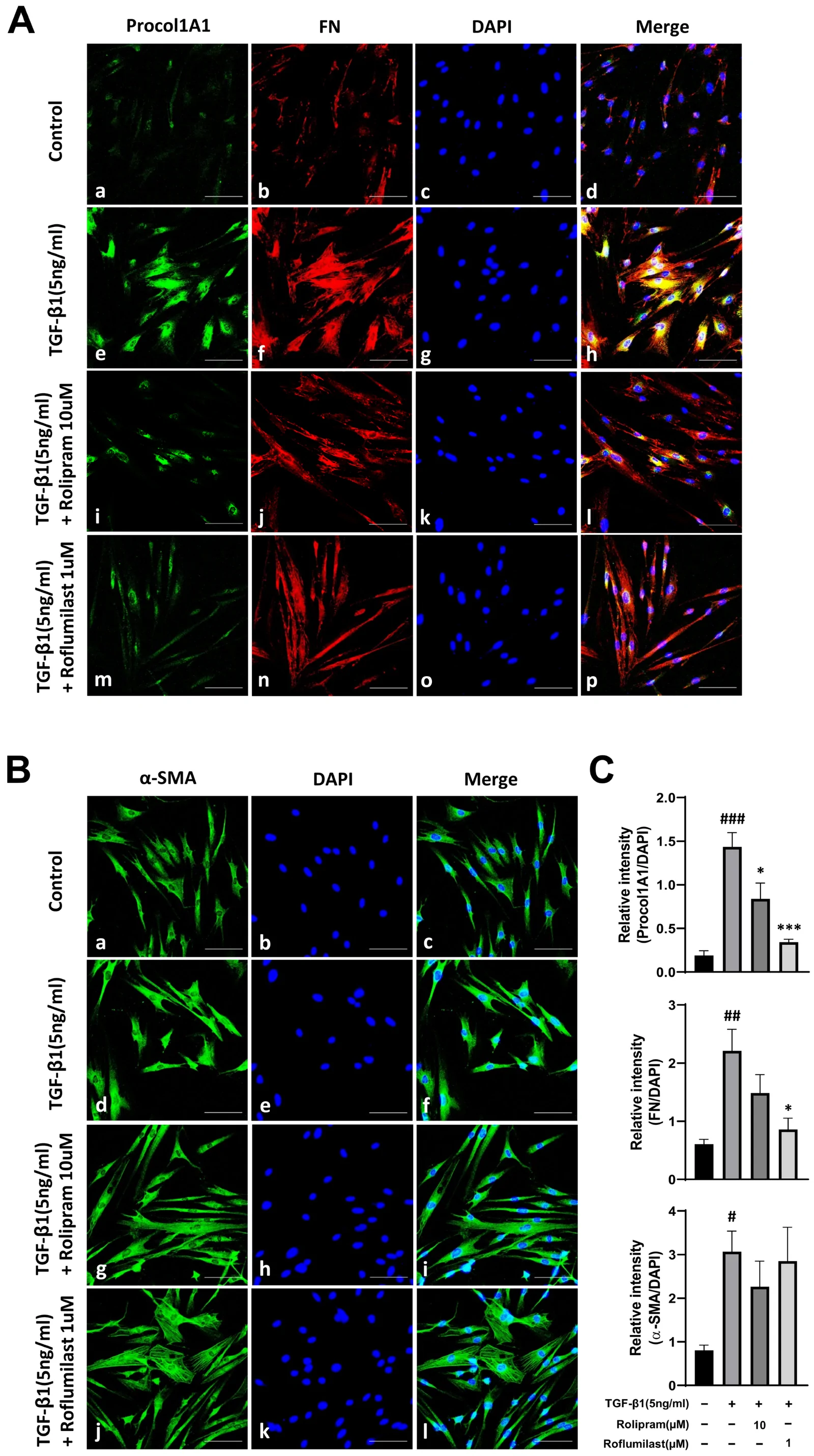

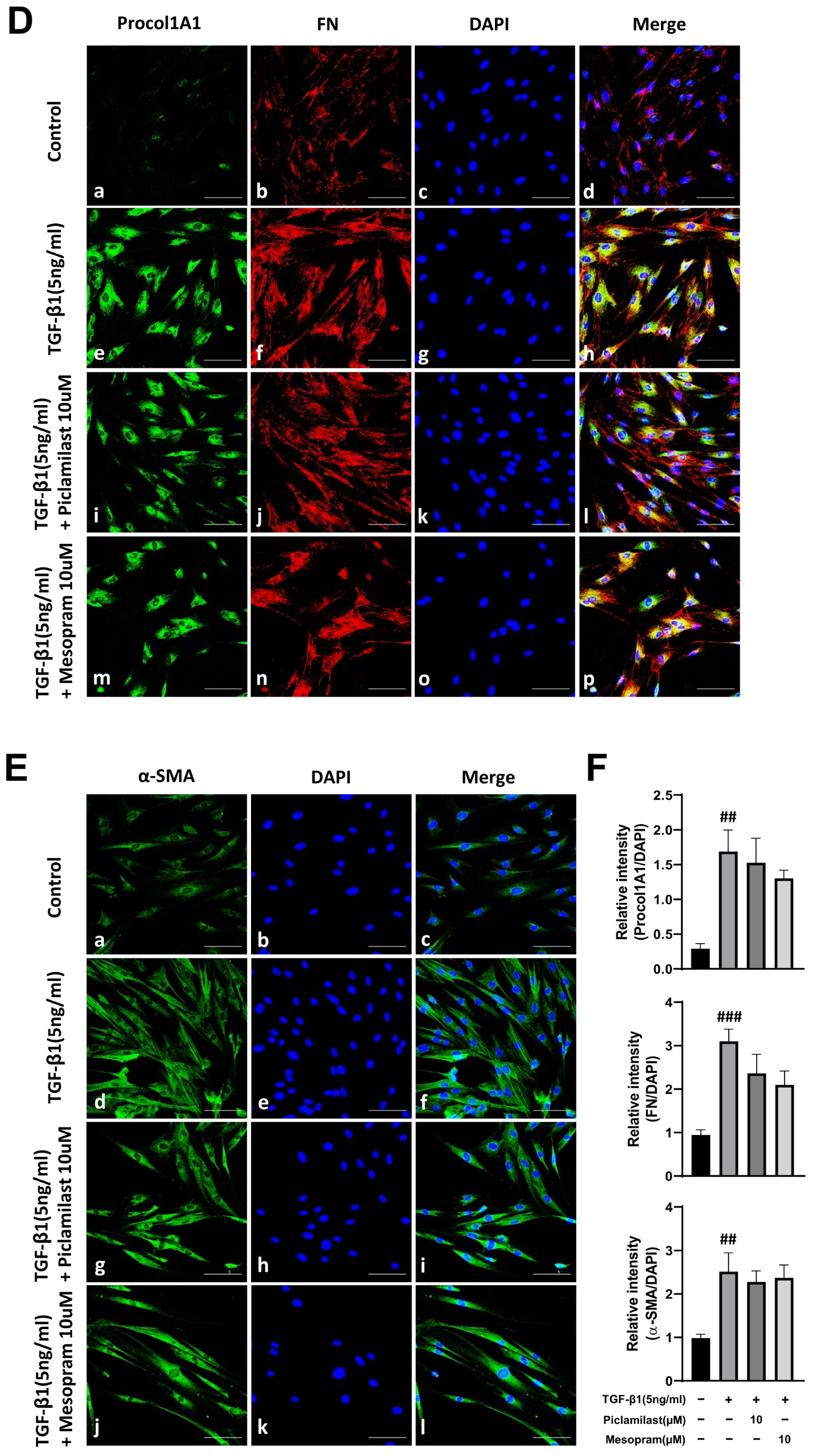
Co-culture with PDE4 inhibitors suppresses TGF-β1-induced Procol1A1, FN, and α-SMA expression in HIMFs. (**A**–**F**) HIMFs were treated with TGF-β1 (5 ng/mL) and co-cultured with or without PDE4 inhibitors for 48 h then stained with Procol1A1, FN, and α-SMA antibodies and counterstained with DAPI. (**A**,**D**) (a–d) no treatment; (e–h) treatment with TGF-β1; (i–p) treatment with TGF-β1 and the indicated PDE4 inhibitors. (**B**,**E**), (a–c) no treatment; (d–f) treatment with TGF-β1; (g–l) treatment with TGF-β1 and the indicated PDE4 inhibitors. The fluorescence intensity ratio of Procol1A1, FN, and α-SMA to DAPI was quantified and plotted using ImageJ software (version 1.50i; National Institutes of Health, Bethesda, MD, USA) as described in Section 4. Data are expressed as the means ± SEM. ^#^ *p* < 0.05, ^##^ *p* < 0.01, and ^###^ *p* < 0.001 versus the control; * *p* < 0.05, *** *p* < 0.001 versus the TGF-β1 treatment only (ANOVA w/Tukey). Scale bar = 100 μm; original magnification, ×200.

Immunostaining results revealed a substantial increase in Procol1A1 and FN staining intensity in TGF-β1-treated HIMFs (Figure 3A,C,D,F). Treatment with roflumilast (1 µM) or rolipram (10 µM) in the presence of TGF-β1 reduced the staining intensity of Procol1A1 and FN (Figure 3A,C). In contrast, immunostaining of α-SMA in TGF-β1-treated HIMFs revealed robust and densely stained actin stress fibers, but the staining intensity was not significantly altered by roflumilast or rolipram (Figure 3B,C). Moreover, piclamilast or mesopram (10 µM) did not substantially affect the staining patterns of Procol1A1, FN, or α-SMA (Figure 3D–F).

### 2.3. PDE4 Inhibitors’ Impact on MRTF/SRF Signaling in Human Intestinal Myofibroblasts

**Figure 4:**
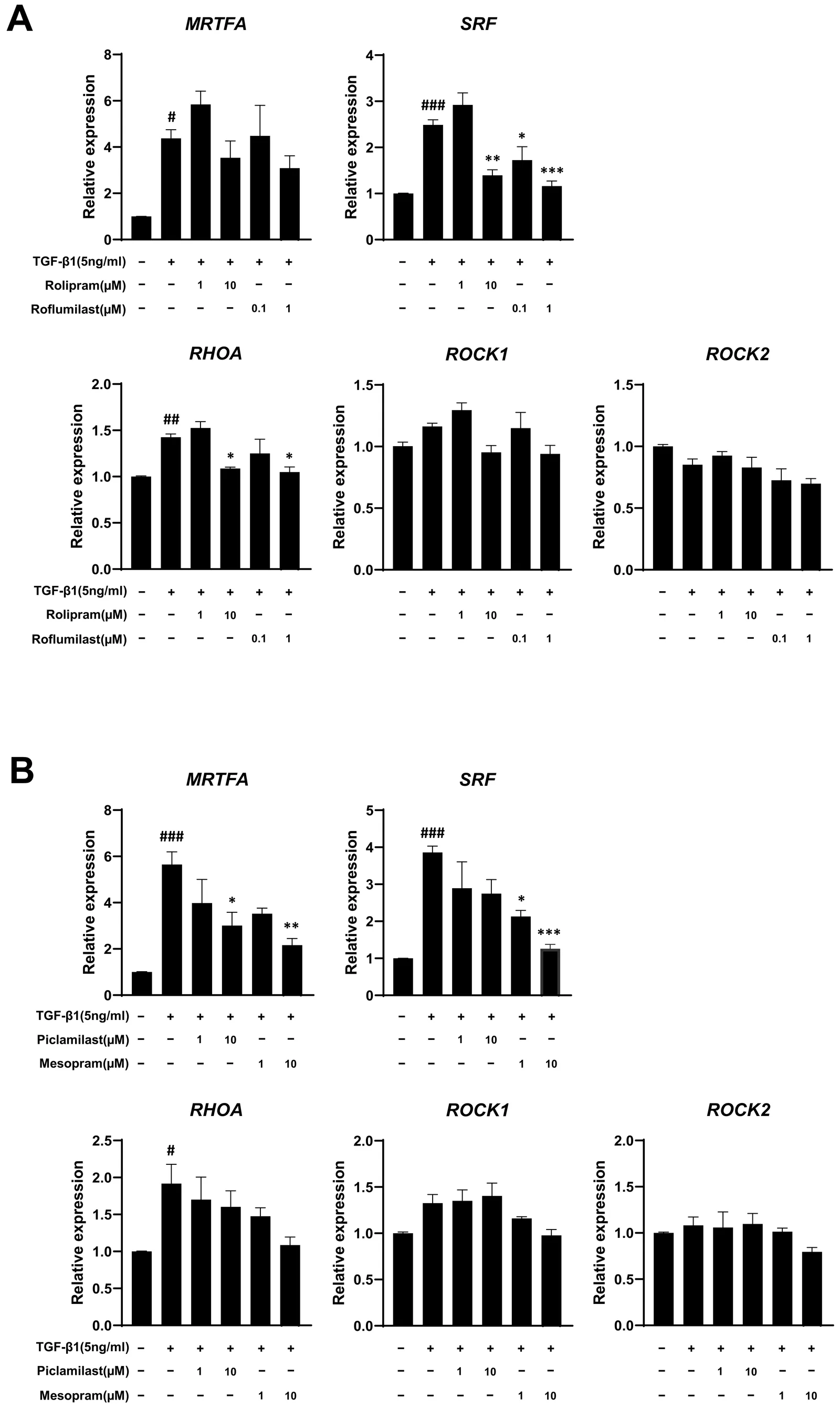
PDE4 inhibitors suppress MRTF/SRF signaling in HIMFs. HIMFs were treated with TGF-β1 (5 ng/mL) and co-cultured with or without PDE4 inhibitors for 24 h. (**A**,**B**) RT-qPCR analysis of the relative mRNA expression of *MRTFA*, *SRF*, *RHOA*, *ROCK1*, and *ROCK2*. The data were normalized to *GAPDH* expression and expressed as relative values compared to the control (rolipram, *n* = 4; roflumilast, *n* = 4; piclamilast, *n* = 3; and mesopram, *n* = 3). Data are expressed as the means ± SEM. ^#^ *p* < 0.05, ^##^ *p* < 0.01, and ^###^ *p* < 0.001 versus the control; * *p* < 0.05, ** *p* < 0.01, and *** *p* < 0.001 versus the TGF-β1 treatment only (ANOVA w/Tukey).

**Figure 5:**
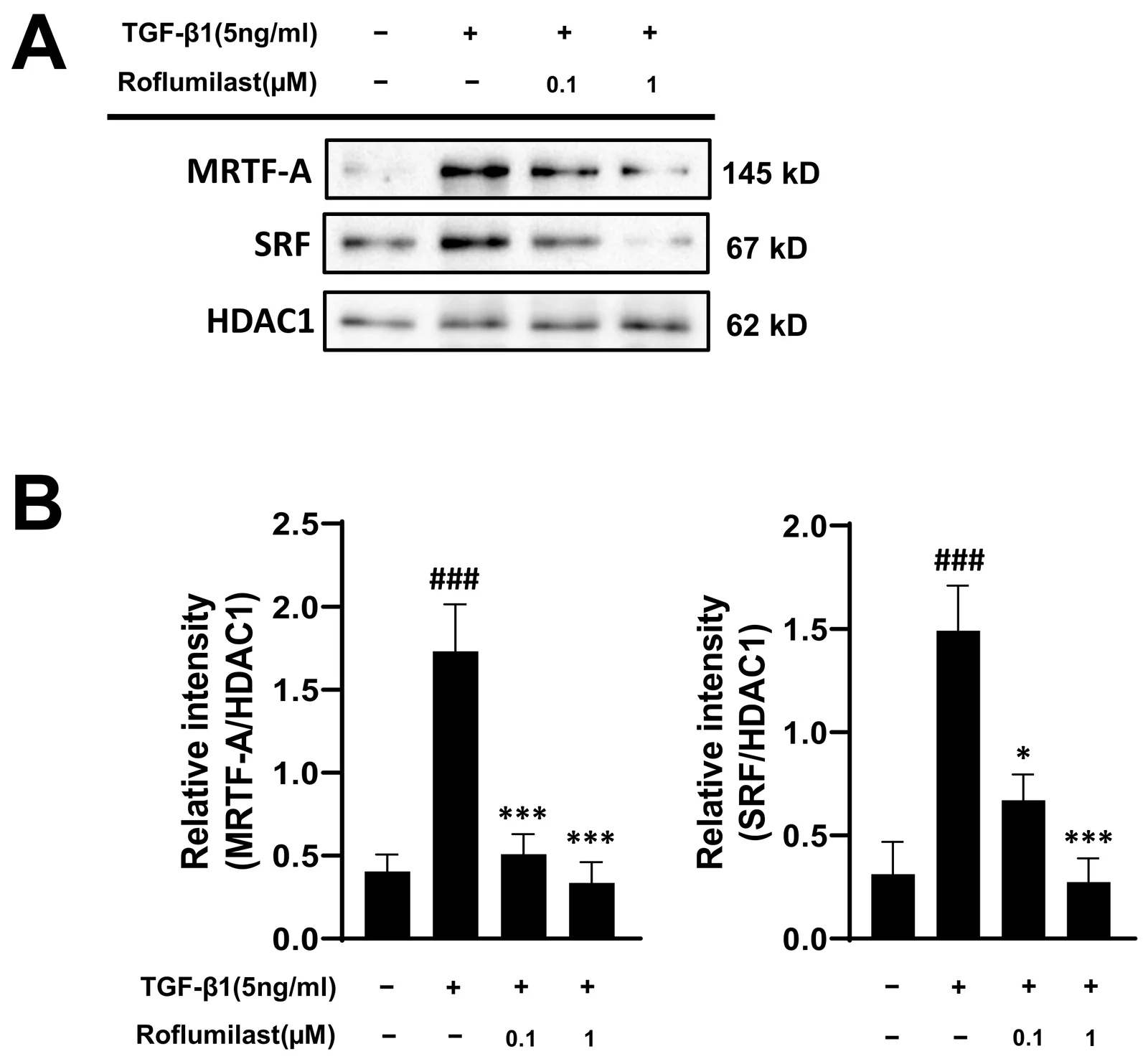
Roflumilast suppresses TGF-β1-induced nuclear expression of MRTF-A and SRF in HIMFs. HIMFs were treated with TGF-β1 (5 ng/mL) and co-cultured with or without roflumilast for 48 h. (**A**) Representative Western blots show the protein expression of MRTF-A and SRF (MRTF-A and SRF in the nuclear extracts, with HDAC1 as a loading control). (**B**) Quantitation of MRTF-A and SRF using Western blot analyses (*n* = 5). Data are expressed as the means ± SEM. ^###^ *p* < 0.001 versus the control; * *p* < 0.05, *** *p* < 0.001 versus the TGF-β1 treatment only (ANOVA w/Tukey).

**Figure 6:**
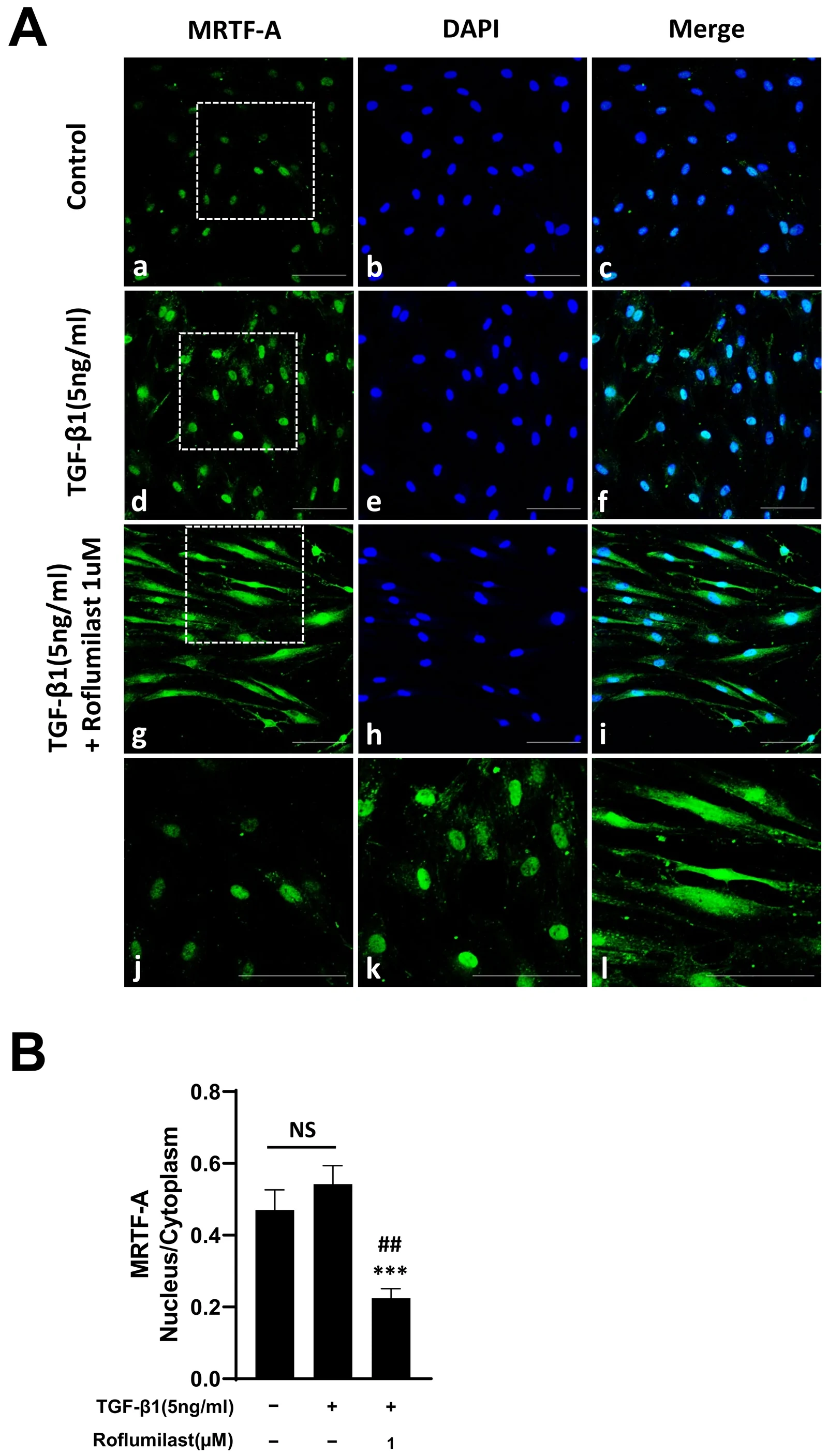
Roflumilast inhibits TGF-β1-induced nuclear translocation of MRTF-A in HIMFs. HIMFs were treated with TGF-β1 (5 ng/mL), co-cultured with or without roflumilast for 48 h, stained with MRTF-A antibody, and counterstained with DAPI. (**A**) (a–c) no treatment; (d–f) treatment with TGF-β1; (g–i) treatment with TGF-β1 and roflumilast; (j–l) enlarged images of the region (within the white box of A, D, and G, each). (**B**) The nuclear-to-cytoplasmic ratio of MRTF-A was determined for each condition as described in Section 4. Data are expressed as the means ± SEM. ^##^ *p* < 0.01 versus the untreated control; *** *p* < 0.001 versus the TGF-β1 treatment only (ANOVA w/Tukey). NS, not significant (Mann–Whitney U test); Scale bar = 100 μm; original magnification, ×200.

We further explored whether PDE4 inhibitors could reduce the TGF-β1-induced expression of Procol1A1, FN, and α-SMA in HIMFs by targeting MRTF/SRF signaling cascade. Rolipram (10 µM) and roflumilast (0.1 µM and 1 µM) significantly reduced *SRF* mRNA expression and exhibited a decreasing trend in *MRTFA* mRNA expression without statistical significance (Figure 4A). Piclamilast (10 µM) significantly decreased TGF-β1-induced MRTFA expression and displayed a decreasing trend in *SRF* mRNA expression (Figure 4B). Mesopram (1 µM) tended to attenuate TGF-β1-induced *MRTFA* expression and significantly reduced *SRF* expression, whereas mesopram (10 µM) significantly reduced the expression of both *MRTFA* and *SRF*. Additionally, we investigated key upstream signaling molecules, including RhoA, ROCK1, and ROCK2. Rolipram (10 µM) and roflumilast (1 µM) significantly reduced *RHOA*, tended to decrease *ROCK1*, and did not affect *ROCK2* expression (Figure 4A). Piclamilast exhibited a decreasing trend in *RHOA* mRNA expression without significant effects on *ROCK1* or *ROCK2* (Figure 4B). Mesopram (10 µM) tended to reduce the TGF-β1-induced mRNA expression of *RHOA*, *ROCK1*, and *ROCK2* in HIMFs. Consistent with their impact on mRNA expression, roflumilast (0.1 µM and 1 µM) significantly suppressed TGF-β1-induced protein expression of MRTF-A and SRF in the nuclear fraction of HIMFs (Figure 5A,B and Figure S2). Moreover, roflumilast significantly reduced MRTF-A nuclear localization in TGF-β1-stimulated cells (Figure 6A,B). These findings suggest that roflumilast may inhibit MRTF/SRF signaling in the HIMFs.

### 2.4. PDE4 Inhibitors’ Suppression of TGF-β1-Induced Phosphorylation of Smad2

**Figure 7:**
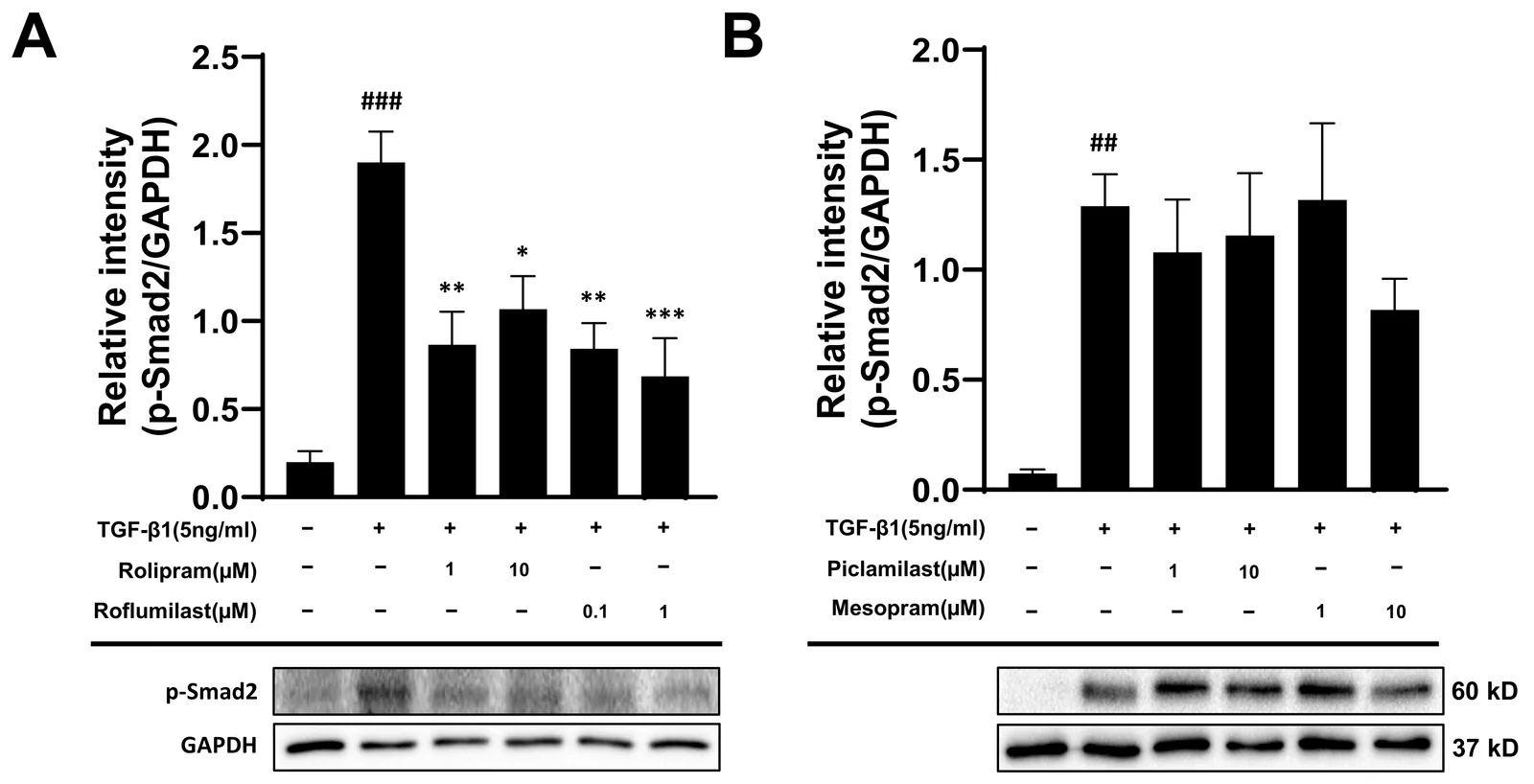
PDE4 inhibitors suppress TGF-β1-induced Smad2 phosphorylation. HIMFs were treated with TGF-β1 (5 ng/mL) and co-cultured with or without PDE4 inhibitors for 48 h. (**A**,**B**) Representative Western blots show the protein expression of phosphorylated Smad2 (p-Smad2), with GAPDH as a loading control. Quantitation of p-Smad2 using Western blot analyses (rolipram, *n* = 5; roflumilast, *n* = 5; piclamilast, *n* = 8; and mesopram, *n* = 8). Data are expressed as the means ± SEM. ^##^ *p* < 0.01 and ^###^ *p* < 0.001 versus the control; * *p* < 0.05, ** *p* < 0.01 and *** *p* < 0.001 versus the TGF-β1 treatment only (ANOVA w/Tukey).

We explored the involvement of classical Smad-dependent TGF-β pathways in mediating anti-fibrotic effects of PDE4 inhibitors in HIMFs. Both roflumilast and rolipram significantly reduced TGF-β1-induced Smad2 phosphorylation (Figure 7A and Figure S3). However, piclamilast did not decrease p-Smad2 expression, and mesopram exhibited a decreasing trend without statistical significance (Figure 7B and Figure S3). These observations suggest anti-fibrogenic effects of PDE4 inhibitors, particularly roflumilast and rolipram may be mediated via Smad-dependent mechanisms. To determine whether Smad2 activation was sustained during prolonged TGF-β1 stimulation, we performed a time-course analysis. Phosphorylated Smad2 remained consistently elevated from 0.5 to 48 h following TGF-β1 treatment (Supplementary Figure S6), supporting the use of the 48 h time point for evaluation of sustained fibrogenic signaling.

### 2.5. Validation of the Anti-Fibrotic Effect of Roflumilast in Crohn’s Disease-Derived Human Intestinal Myofibroblasts

To evaluate whether the anti-fibrotic effect of roflumilast is maintained in a disease-relevant cellular model, we performed additional validation experiments using primary human intestinal myofibroblasts isolated from inactive, non-fibrotic Crohn’s disease lesions (HIMF-CD). Similarly to normal HIMFs, TGF-β1 stimulation significantly increased the mRNA expression of *COL1A1*, *FN1*, and *ACTA2*, whereas treatment with roflumilast significantly attenuated these increases (Supplementary Figure S7A). Consistently, Western blot analysis demonstrated that roflumilast markedly reduced the TGF-β1-induced protein expression of Procol1A1, FN, and α-SMA in HIMF-CD cells (Supplementary Figure S7B–D). These findings indicate that the anti-fibrotic activity of roflumilast is preserved in patient-derived intestinal myofibroblasts from Crohn’s disease, supporting its potential therapeutic relevance in intestinal fibrosis.

## 3. Discussion

The aim of this study was to investigate whether PDE4 inhibitors (rolipram, roflumilast, piclamilast, and mesopram) inhibit the fibrogenic activation of HIMF, and to identify the intracellular mechanisms involved in the inhibitory effect. All PDE4 inhibitors significantly reduced TGF-β1-induced mRNA expression of collagen1A1 (*COL1A1*) and *FN*, with mesopram additionally attenuating *ACTA2* mRNA expression. However, at the protein level, only roflumilast significantly decreased Procol1A1 and FN levels. Roflumilast, rolipram, and mesopram notably reduced *SRF* mRNA expression, whereas piclamilast and mesopram significantly inhibited *MRTFA* mRNA expression. Notably, roflumilast showed the strongest inhibitory effect, markedly decreasing the expressions of both MRTF-A and SRF at the protein level, substantially reducing the nuclear localization of MRTF-A in HIMFs. Additionally, rolipram and roflumilast significantly mitigated TGF-β1-induced phosphorylation of Smad2, indicating a Smad-dependent anti-fibrotic action. These findings suggest that PDE4 inhibitors, especially roflumilast, effectively attenuate the TGF-β1-induced fibrogenic activation in HIMFs by targeting both the MRTF/SRF and Smad-dependent pathways, highlighting their value for further preclinical investigation in intestinal fibrosis. Importantly, the anti-fibrotic effect of roflumilast was further confirmed in primary intestinal myofibroblasts isolated from patients with Crohn’s disease. Although these cells were obtained from inactive, non-fibrotic lesions, roflumilast consistently suppressed TGF-β1-induced expression of profibrotic markers at both the mRNA and protein levels. These findings extend our observations beyond normal HIMFs and suggest that the anti-fibrotic activity of roflumilast is preserved in patient-derived cells, thereby enhancing the translational relevance of the present study.

Recent research has increasingly recognized the therapeutic potential of PDE4 inhibitors in mitigating fibrosis across various organs. Among first-generation PDE4 inhibitors, rolipram stands out for its significant efficacy in reducing fibrosis in multiple organs [23,24,25]. For instance, in bleomycin-induced lung fibrosis models in rodents, rolipram effectively decreased fibrosis scores, reduced hydroxyproline content, and lowered serum tumor necrosis factor-alpha (TNF-α) levels, highlighting its anti-fibrotic effects [23]. In systemic sclerosis mouse models, rolipram not only halted the progression of chronic fibrosis but also reversed established fibrosis [24]. In gastrointestinal fibrosis, rolipram suppressed collagen and TGF-β1 expression in a rat model of trinitrobenzene sulfonic acid-induced colitis [28]. Moreover, in a murine cecal abrasion model, rolipram showed potential in preventing postoperative intra-abdominal adhesions by inhibiting fibrotic reactions [30]. Second-generation PDE4 inhibitors, such as roflumilast and apremilast, have also shown promise. Roflumilast reduced TGF-β1-induced collagen I and FN expression in human airway smooth muscle cells [31], and inhibited liver fibrosis induced by diethyl nitrosamine in rats, as evidenced by reduced hydroxyproline deposition [32]. In models of diabetic nephropathy, roflumilast significantly improved renal damage caused by a high-fat diet, reducing collagen deposition and the expression of FN and collagen IV [33]. Apremilast also demonstrated effectiveness in reducing colonic fibrosis in a dextran sulfate sodium (DSS)-induced colitis mouse model [34] and regulated the cAMP-predominant PKA–CREB signaling pathway in a chronic DSS-induced murine colitis model, thereby ameliorating intestinal fibrosis [29].

In our current study, PDE4 inhibitors significantly inhibit TGF-β1-induced fibrogenic activation in HIMFs, as evidenced by decreased expression of ECM proteins (Procol1A1, FN) and contractile protein (α-SMA). PDE4 inhibitors have previously been shown to inhibit fibroblast-to-myofibroblast differentiation in vitro, decreasing ECM and α-SMA expression. For instance, piclamilast suppressed TGF-β-mediated fibroblast-to-myofibroblast differentiation [35], while roflumilast inhibited both fibroblast chemotaxis towards FN and TGF-β-stimulated fibroblast contraction of collagen gels [36]. Moreover, TGF-β1-induced epithelial mesenchymal transition (EMT), a critical event in organ fibrosis, is modulated by PDE4 and cAMP-PDE4 activity. The inhibition of PDE4 by rolipram significantly inhibited EMT by reducing collagen I and FN expression [37]. In the present study, mesopram and piclamilast demonstrated significant anti-fibrogenic effects at the RNA level but not at the protein level. These discrepancies might result from time-dependent differences in gene expression regulation, as our measurements at 24 and 48 h provide limited snapshots. Additionally, there are regulatory complexities at the transcriptional, post-transcriptional, and post-translational levels [38].

The consequence of PDE4 inhibition is elevation of intracellular cAMP levels, resulting in activation of the two principal cAMP effector pathways, PKA and Epac. This PDE4–cAMP–PKA/Epac signaling axis has been well established in numerous cell types and is considered the central mechanism underlying the biological effects of PDE4 inhibitors. Through these downstream effectors, elevated cAMP regulates multiple profibrotic signaling pathways, including RhoA/ROCK/MRTF/SRF signaling and TGF-β/Smad signaling. Recent studies have highlighted the importance of Rho/ROCK/Actin/MRTF/SRF signaling in intestinal fibrosis [39,40,41]. The Rho/ROCK pathway is inhibited by elevated intracellular cAMP via cAMP/PKA signaling [27,42,43]. Elevated cAMP inhibits RhoA activity, reducing actin-myosin contraction and stress fiber formation [44]. PDE4 inhibitors may exert anti-fibrotic effects by increasing cAMP levels, thereby inhibiting Rho/ROCK/MRTF/SRF signaling. Rolipram has previously demonstrated inhibitory effects on RhoA in a cAMP-dependent manner [45], and apremilast has shown anti-inflammatory activity via inhibition of ROCK2 [46]. Consistent with these findings, our data suggest PDE4 inhibitors modulate Rho/ROCK/MRTF/SRF signaling. In our study, roflumilast, rolipram, and mesopram significantly reduced *SRF* mRNA expression, while piclamilast and mesopram significantly inhibited *MRTFA* mRNA expression. Notably, roflumilast significantly reduced protein expression and nuclear localization of MRTF-A and SRF in TGF-β1-stimulated HIMFs. To further investigate upstream modulation of MRTF/SRF signaling, we assessed the expression of *RHOA*, *ROCK1*, and *ROCK2*. Rolipram (10 µM) and roflumilast (1 µM) significantly reduced TGF-β1-induced *RHOA* expression and showed a decreasing trend in *ROCK1*, whereas piclamilast and mesopram exhibited only non-significant decreasing trends. Roflumilast primarily exerts its anti-fibrotic effects by targeting the TGF-β pathway at a more downstream level. This involves the inhibition of the MRTF/SRF pathway, including the modulation of MRTF nuclear localization, as the central mechanism of action.

The present study demonstrates that both rolipram and roflumilast significantly reduce TGF-β1-induced Smad2 phosphorylation, suggesting the involvement of Smad-dependent mechanisms in their anti-fibrotic effects. TGF-β1-induced fibrogenic activation in myofibroblasts is mediated through Smad-dependent and Smad-independent pathways [11,39,47,48]. Specifically, Smad-dependent signaling involves phosphorylation of Smad2 and Smad3, leading to their nuclear translocation and activation of pro-fibrotic gene transcription [47,48,49]. Consistent with our findings, rolipram has been previously shown to inhibit Smad signaling and TGF-β1-regulated genes associated with airway remodeling [50], while apremilast demonstrated anti-fibrotic effects by suppressing TGF-β1-induced Smad2/3 and Erk1/2 phosphorylation in human skin fibroblasts [24,51]. However, rolipram did not inhibit TGF-β1-induced epithelial–mesenchymal transition (EMT) via Smad phosphorylation in human alveolar epithelial cells, suggesting cell-type-specific differences [37].

It should be noted that the present study evaluated Smad2 phosphorylation and MRTF-A nuclear localization after 48 h of continuous TGF-β1 stimulation, rather than during the early signaling phase. Although Smad2 phosphorylation is generally initiated within minutes after TGF-β stimulation and MRTF-A rapidly translocates to the nucleus within approximately one hour, our objective was to investigate the signaling events associated with sustained fibrogenic activation under the same experimental conditions used to assess the downstream fibrotic phenotype (Procol1A1, FN, and α-SMA). Importantly, in our HIMF model, Smad2 activation was not transient but remained persistently elevated throughout TGF-β1 stimulation. As shown in the time-course analysis (Supplementary Figure S6), phosphorylation of Smad2 was maintained from 0.5 to 48 h after TGF-β1 treatment. These findings are consistent with our previous studies using the identical HIMF model, in which TGF-β1-induced Smad2/3 phosphorylation remained elevated for at least 24–48 h and sustained nuclear accumulation of MRTF-A was observed during prolonged stimulation [11,52]. Therefore, the 48 h time point reflects an ongoing profibrotic signaling state rather than a delayed compensatory response. Furthermore, the suppression of p-Smad2 and nuclear MRTF-A by roflumilast at 48 h closely paralleled the reductions in ECM proteins and α-SMA under identical experimental conditions, supporting the conclusion that these changes represent genuine inhibition of sustained profibrotic signaling rather than nonspecific secondary effects.

**Figure 8:**
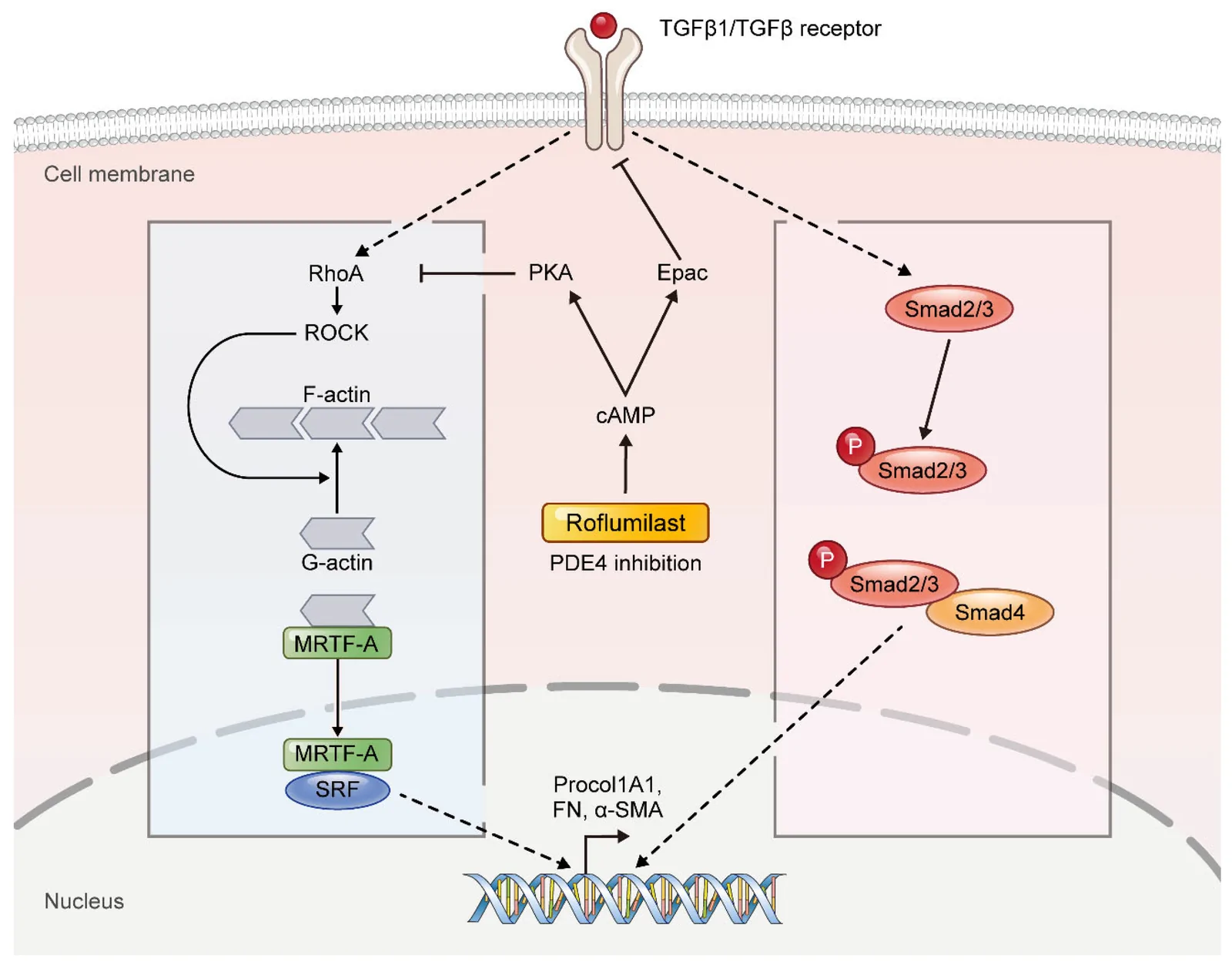
Roflumilast-mediated PDE4 inhibition increases intracellular cAMP, which attenuates TGF-β1-induced fibrosis through two distinct cAMP effectors acting on separate branches of TGF-β signaling. (1) Smad-independent branch (**left**): cAMP activates PKA, which inhibits RhoA activity and thereby suppresses the downstream ROCK/F-actin polymerization cascade, preventing MRTF-A nuclear translocation and MRTF-A/SRF expression. (2) Smad-dependent branch (**right**): roflumilast reduced TGF-β1-induced Smad2 phosphorylation in HIMFs. This effect is proposed to be mediated by the cAMP effector Epac, which has been reported to interact with the TGF-β type I receptor (ALK5) and inhibit Smad2/3 phosphorylation, nuclear translocation, and complex formation with Smad4.

Traditionally, anti-fibrotic effects of elevated cAMP have been attributed primarily to the activation of the PKA/CREB signaling pathway, which inhibits Smad2/3 binding to CREB-binding protein (CBP), thereby reducing pro-fibrotic transcriptional activity [53,54]. However, accumulating evidence suggests that PDE4 inhibitor-mediated inhibition of Smad2/3 phosphorylation may also involve CREB-independent mechanisms, particularly through Epac (Exchange protein directly activated by cAMP). Epac is a guanine nucleotide exchange factor for Rap1, acting as an alternative cAMP effector independently of PKA, and has been implicated in anti-fibrotic effects by inhibiting Smad2 phosphorylation via direct interactions with the TGF-β receptor type I (TGFβR1/ALK5) [27,50,51,55]. Thus, further studies are required to clarify whether the inhibition of Smad2 phosphorylation observed with rolipram and roflumilast in this study occurs primarily through Epac-dependent rather than classical PKA/CREB-dependent mechanisms. We propose that roflumilast exerts anti-fibrotic effects through two complementary cAMP-dependent mechanisms. First, activation of the cAMP/PKA axis suppresses RhoA/ROCK/F-actin signaling, thereby preventing MRTF-A nuclear translocation and reducing MRTF/SRF-mediated profibrotic transcription. Second, activation of the cAMP/Epac pathway may inhibit ALK5-dependent Smad2/3 phosphorylation and nuclear translocation, thereby attenuating canonical TGF-β signaling. A schematic representation of these proposed mechanisms is shown in Figure 8.

In the present study, roflumilast exhibits more pronounced anti-fibrogenic efficacy in HIMFs compared to other PDE4 inhibitors, including rolipram, piclamilast, and mesopram. While direct comparisons of PDE4 inhibitors’ anti-fibrotic effects within the same cell types remain limited, roflumilast demonstrated superior potency, particularly at the protein level. This enhanced potency might result from roflumilast’s superior ability to elevate intracellular cAMP levels, approximately twice as effectively as other PDE4 inhibitors such as rolipram, apremilast, and cilomilast [56]. Elevated cAMP is known to mediate both anti-inflammatory and anti-fibrogenic effects, including inhibition of ECM protein synthesis and reduction in fibroblast proliferation [57,58]. Interestingly, despite these observed differences in cellular potency, in vivo studies have reported comparable anti-fibrotic efficacy among PDE4 inhibitors, although these findings were not specifically from intestinal fibrosis models. For instance, roflumilast and rolipram exhibited similar effectiveness in reducing lung collagen deposition in a pulmonary fibrosis model [59]. Similarly, rolipram and apremilast exhibited equally robust anti-fibrotic effects in bleomycin-induced skin fibrosis models, mediated by reduced profibrotic cytokine release from M2 macrophages, subsequently diminishing fibroblast activation and collagen production [24]. Moreover, both rolipram and roflumilast treatments showed comparable anti-fibrotic efficacy in renal fibrosis models by decreasing collagen deposition and increasing metalloproteinase 9 fibrinolytic activity [60].

Importantly, our study found no significant cytotoxicity at effective concentrations of roflumilast, piclamilast, and mesopram, aligning well with previous clinical data. Roflumilast has been clinically utilized for over a decade primarily for COPD and asthma, demonstrating a favorable safety and tolerability profile in large-scale randomized controlled trials for COPD, psoriasis, and atopic dermatitis [28,61,62,63]. In contrast, rolipram, despite effectively ameliorating chronic colitis and intestinal fibrosis, has limited clinical utility due to dose-limiting gastrointestinal adverse events [28,62]. Thus, roflumilast may have value for further preclinical investigation in intestinal fibrosis, given its potent anti-fibrotic effects and favorable safety profile.

The partial inhibition (~50%) of fibrotic markers observed with selective PDE4 blockade suggests potential contributions from other PDE isoforms, particularly PDE3. Previous studies have indicated that PDE3 inhibitors significantly reduce fibrosis in cardiac and pulmonary fibrosis models, displaying synergistic effects when combined with PDE4 inhibitors. Hence, targeting multiple PDE isoforms may provide enhanced anti-fibrotic efficacy [64,65].

We further explored the variability in anti-fibrotic effects among PDE4 inhibitors, despite similar PDE4 inhibition efficiencies. Notably, all four inhibitors were applied at concentrations well above their inhibitory constants, at which catalytic PDE4 inhibition would be near-complete, and because these competitive inhibitors act at the conserved catalytic site, they display little selectivity among the PDE4A–D subtypes. Nevertheless, the individual inhibitors differ in their physicochemical properties and intracellular disposition, which can influence how effectively each drug reaches the discrete subcellular compartments in which specific PDE4 isoforms are anchored [66,67]. Because these compartmentalized PDE4 isoforms govern spatially restricted, local cAMP pools through their N-terminal targeting regions, differential access to distinct PDE4-containing microdomains may translate into different local elevations of cAMP and, consequently, distinct downstream biological responses even when bulk catalytic inhibition is comparable [67,68]. Accordingly, the superior activity of roflumilast is unlikely to reflect catalytic potency or binding affinity alone [69]; it may instead arise from more effective functional engagement of the relevant cAMP–PDE4 compartments, together with its stronger PGE2-dependent autocrine amplification (Supplementary Figure S5). The literature indicates that PDE4 inhibitor-mediated anti-fibrotic effects are dependent on extracellular PGE2 induction, disrupted by PGE2 synthesis inhibitors like indomethacin [70,71]. Prostaglandin E2 is known to block nuclear localization of MRTF, a key regulator of myofibroblast differentiation [70], and prostaglandin E receptor 2 (EP2) receptor-mediated signaling contributes significantly to these anti-fibrotic processes [71]. Our experiments using ELISA revealed variability in PGE2 secretion among PDE4 inhibitors, with roflumilast significantly enhancing extracellular PGE2 release, supporting the critical role of the PGE2/AC/cAMP signaling pathway (Supplementary Figure S5).

Rolipram and roflumilast significantly reduced TGF-β1-induced Smad2 phosphorylation (~60%), whereas piclamilast showed minimal efficacy. These differences likely arise from variations in binding affinities, PDE4 isoform selectivity, and modulation by other intracellular signaling molecules [17,66]. The intracellular environment—including additional modulators such as PGE2, which activates the cAMP/PKA pathway through EP2/EP4 receptors, and other phosphodiesterases like PDE3 that degrade cAMP—can further modulate the efficacy of PDE4 inhibitors [67,68]. Additionally, factors such as cell type, passage number, PDE4 isoform expression levels, and experimental conditions (e.g., serum concentration, cell density) significantly influence inhibitor effectiveness. These findings underscore the necessity of considering isoform-specific interactions and broader regulatory networks to fully understand the differential effects of PDE4 inhibitors on fibrotic signaling pathways.

There are several limitations in our study. First, the mechanism underlying the superior anti-fibrotic effect of roflumilast at the protein level was not directly investigated. Although roflumilast induced the greatest PGE2 secretion among the PDE4 inhibitors tested (Supplementary Figure S5), and previous studies have suggested that endogenous PGE2 may enhance the anti-fibrotic actions of PDE4 inhibition through autocrine/paracrine signaling, our data do not establish such a mechanism [36,69,72,73]. In particular, PGE2 was measured at a single time point, and no experiments were performed to determine whether the observed suppression of profibrotic protein expression was dependent on PGE2 signaling. Therefore, the possibility that enhanced PGE2 production contributed to the greater efficacy of roflumilast remains speculative. Additional studies incorporating time-course analyses, dose–response experiments, pathway-specific inhibition, and direct assessment of collagen secretion will be required to clarify the mechanisms responsible for the differential anti-fibrotic effects observed among PDE4 inhibitors. Second, the present study is that the involvement of the MRTF-A/SRF and Smad2 signaling pathways was primarily inferred from expression changes and nuclear localization analyses, without direct intervention or rescue experiments in the current experimental setting. Although our findings consistently demonstrated that roflumilast attenuated TGF-β1-induced fibrotic responses in HIMFs in parallel with suppression of MRTF-A/SRF signaling, a definitive causal relationship was not directly tested in this study. However, our previous work using the selective MRTF-A/SRF inhibitor CCG-100602 in the identical cell model (HIMFs) and under the same fibrogenic stimulus (TGF-β1) demonstrated that pharmacological inhibition of MRTF-A/SRF signaling abolished the induction of profibrotic markers, including procollagen 1A1, fibronectin, and α-SMA, thereby establishing the functional necessity of this pathway in intestinal fibrogenesis [11]. The anti-fibrotic phenotype observed following MRTF-A/SRF inhibition closely resembles that induced by roflumilast in the present study. Nevertheless, future studies incorporating pathway-specific inhibition, genetic manipulation, or rescue approaches will be necessary to further confirm the direct mechanistic contribution of MRTF-A/SRF and Smad2 signaling to the anti-fibrotic effects of roflumilast.

In conclusion, the absence of effective anti-fibrotic treatments remains a significant challenge in addressing intestinal fibrosis. Among the PDE4 inhibitors examined in this study, roflumilast effectively suppressed TGF-β1-induced fibrogenic activation in HIMFs by targeting Smad-independent and Smad-dependent pathways (Figure 8). PDE4 inhibition by roflumilast elevates intracellular cAMP, which suppresses TGF-β1-induced fibrogenesis through two distinct cAMP effectors acting on separate branches of TGF-β signaling. For the Smad-independent branch, cAMP activates PKA, which phosphorylates RhoA and inhibits the downstream ROCK/F-actin/MRTF-A/SRF cascade. For the Smad-dependent branch, we observed that roflumilast reduced TGF-β1-induced Smad2 phosphorylation by Western blot. We propose that this effect is mediated not by a direct action of roflumilast on Smad2/3, but by the cAMP effector Epac: Epac1 has been reported to physically interact with the type I TGF-β receptor (ALK5) and inhibit Smad2 phosphorylation and transcriptional activation [74], and Epac1 overexpression suppresses TGF-β1-induced collagen synthesis [75]. While the reduction in p-Smad2 is a direct experimental observation, the involvement of Epac as the upstream mediator was not directly tested in this study, and we present the cAMP/Epac–ALK5 link as a putative, literature-derived mechanism. Through these pathways, roflumilast attenuates the transcriptional activation of pro-fibrotic markers, including *COL1A1*, *FN1*, and *ACTA2*.

## 4. Materials and Methods

### 4.1. Reagents

Recombinant human transforming growth f actor-β1 (TGF-β1) was sourced from R&D Systems (Minneapolis, MN, USA). The PDE4 inhibitors rolipram and roflumilast were procured from Sigma-Aldrich (St. Louis, MO, USA), while piclamilast and mesopram were obtained from Tocris Bioscience (Minneapolis, MN, USA).

### 4.2. Human Intestinal Myofibroblast Isolation and Culture

HIMFs were isolated and cultured using a method adapted from previously described protocols [11,76]. In brief, HIMFs were extracted from outgrowths of finely chopped colonic mucosa explants, which were placed in etched polystyrene flasks filled with HIMF growth medium. This medium contained Dulbecco’s Modified Eagle’s Medium/high glucose (Hyclone, Logan, UT, USA), supplemented with fetal bovine serum, L-glutamine, HEPES, penicillin, streptomycin, and amphotericin B (all from Lonza, Walkersville, MD, USA). Cells between passages 6 and 10 at 80% confluence were used for experiments. The phenotype of HIMFs (passage 5) was characterized using immunofluorescence microscopy, confirming that the cells were α-smooth muscle actin-positive (70–80%), Vimentin-positive (100%), and Desmin-negative (positive rate <5%) (Supplementary Figure S4). The characterization of HIMFs was performed following previously established methods [77].

HIMFs were sourced from histologically normal colon segments obtained post-surgery from colorectal cancer patients. These segments were excised by a pathologist post-surgery from areas close to the proximal resection margin. This study adheres to the Declaration of Helsinki and was approved by Institutional Review Board of the CHA Bundang Medical Center, Korea (Building a prospective/retrospective cohort of human derived materials and clinical data for development of new microRNA biomarkers in inflammatory bowel disease; Approval number: CHAMC 2020-07-021-017; Date of approval: 14 September 2020; Date of approval end: 13 June 2021). During the IRB approval period, we isolated HIMF from colorectal specimens obtained from patients who underwent colonoscopy (with written consent). Written informed consent for specimens was obtained from all adult participants. For the validation experiments, primary intestinal myofibroblasts were additionally isolated from surgically resected, inactive non-fibrotic intestinal tissues of patients with Crohn’s disease (HIMF-CD) under the same IRB approval (CHAMC 2020-07-021-017), with written informed consent obtained from all participants, as detailed in the Supplementary Methods.

### 4.3. Co-Culture of HIMFs with PDE4 Inhibitors

The impact of PDE4 inhibitors on TGF-β1-induced fibrosis in HIMFs was assessed by starving the cells in serum-free medium for 24 h, followed by 30 min pre-treatment with each PDE4 inhibitor and subsequent TGF-β1 (5 ng/mL) exposure for 24 h (RT-qPCR analysis) or 48 h (Western blot analysis).

### 4.4. Cell Viability Assay

Cell viability was assessed using the 3-(4, 5-dimethylthiazol-2-yl)-2,5-diphenyl tetrazolium bromide (MTT) assay (ab211091, Abcam, Cambridge, UK), conducted in accordance with the manufacturer’s protocol. Briefly, HIMFs were seeded in 96-well plates at 0.5 × 10^4^ cells per well in 100 μL of culture medium. Following specific treatments, the existing medium was discarded and replaced with a mixture of 100 µL serum-free medium and MTT reagent in a 1:1 ratio. Cell-free wells were included as background controls. The plates were incubated at 37 °C for 3 h, post which the MTT reagent was aspirated and 150 µL of MTT solvent was added to each well. Subsequent to 15 min of shaking in dark conditions, absorbance was measured at 590 nm using the Multiskan GO microplate reader (ThermoFisher Scientific, Waltham, MA, USA). Variations in cell viability post PDE4 inhibitor treatment were quantified relative to control (vehicle-treated) cells.

### 4.5. RNA Isolation and Real-Time Quantitative PCR (RT-qPCR)

Total RNA extraction from HIMFs was carried out using TRIzol reagent (Ambion, Carlsbad, CA, USA). For cDNA synthesis, 1 µg of RNA was reverse transcribed using the ReverTra Ace qPCR RT Master Mix Kit (TOYOBO, Osaka, Japan), following the manufacturer’s guidelines. The RT-qPCR was performed with a Roche Light Cycler 96 system using the Faster-Start Essential DNA Probes Master (Roche, Basel, Switzerland). Gene expression levels were normalized to GAPDH, using specific primers. Specific primers for the collagen1A1 (Hs00164004_m1), FN (Hs01549976_m1), α-SMA (ACTA2, Hs00426835_g1), Mkl1 (MRTF-A, Hs01090249_g1), SRF (Hs01065256_m1), RhoA (Hs00357608_m1), ROCK1 (Hs01127701_m1), ROCK2 (Hs00178154_m1), and GAPDH (Hs03929097_g1) were obtained from Applied Biosystems (Foster City, CA, USA). The expression level was normalized using GAPDH. The fold changes were calculated by the 2^−ΔΔCt^ method. All RT-qPCR results were validated by three or more independent experiments. All qPCR experiments were performed in triplicate and independently repeated three times to ensure reliability and reproducibility.

### 4.6. Extraction of Nuclear and Cytoplasmic Proteins

Nuclear and cytoplasmic fractions from the HIMFs were isolated using the Nuclear Extraction kit (Millipore, Billerica, MA, USA), as per the manufacturer’s instructions. Briefly, cells were harvested by centrifugation and suspended in Cytoplasmic Lysis Buffer. The solution was vortexed and incubated on ice for 15 min. The cell suspension was then passed through a 27-gauge needle 5 times using a 1 mL syringe followed by centrifugation at 8000× *g* for 20 min at 4 °C. The supernatant was collected as the cytoplasmic extract. The nuclear pellet was suspended with Nuclear Extraction Buffer and passed through a 27-gauge needle 5 times using a 1 mL syringe again. After 1 h of incubation at 4 °C, nuclear suspension was collected by centrifugation at 16,000× *g* for 20 min at 4 °C. Protein concentrations were determined using the bicinchoninic acid assay and subsequently analyzed through Western blotting.

### 4.7. Western Blotting

Protein lysates were prepared using RIPA buffer (Cell Signaling, Beverly, MA, USA) supplemented with proteinase inhibitor cocktail (#P8340, Sigma-Aldrich) and phosphatase inhibitor cocktail (#P0044 and #P5726, Sigma-Aldrich). Fifteen micrograms (15 µg) of protein were loaded per lane. Protein samples, mixed with an equal volume of 5× SDS sample buffer, were boiled for 5 min and resolved on 10% SDS-PAGE gels. Following electrophoresis, proteins were transferred to polyvinylidene difluoride membranes and blocked in 5% nonfat dry milk in Tris-buffered saline with Tween-20 buffer (TBS-T) for 1 h at room temperature. Membranes were incubated overnight at 4 °C with primary antibodies, followed by horseradish peroxidase-conjugated secondary antibodies (1:4000 dilution, GeneTex, Irvine, CA, USA). Detection was performed using the SuperSignal West Pico Chemiluminescence System (Thermo Fisher Scientific) and captured using the ChemiDocTM XRS+ System (Bio-Rad, Hercules, CA, USA). Quantification was performed using ImageJ software (version 1.50i; National Institutes of Health, Bethesda, MD, USA). The primary antibodies used included procollagen1A1 (Procol1A1) (1:5000 dilution, SP1D8, Developmental Studies Hybridoma Bank, Iowa City, IA, USA), FN (1:10,000 dilution, ab2413, Abcam, Cambridge, MA, USA), α-SMA (1:10,000 dilution, A2547, Sigma), phospho-Smad2 (Ser465/467) (1:1000 dilution, #3108, Cell Signaling), RhoA (1:1000 dilution, #sc-418, Santa Cruz Biotechnology, Dallas, TX, USA), GAPDH (1:3000 dilution, #2118, Cell Signaling), Mkl1 (MRTF-A) (1:1000 dilution, 21166-1-AP, ProteinTech, Rosemont, IL, USA), SRF (1:1000 dilution, #5147, Cell Signaling), and HDAC1 (1:1000 dilution, #5356, Cell Signaling).

### 4.8. Immunocytochemistry

Immunofluorescence staining was conducted as previously described [12,30]. To assess the anti-fibrogenic effects of the PDE4 inhibitors, the HIMFs (1.0 × 10^4^ cells/well) were seeded onto the chamber slides (#30108, SPL life Sciences, Pocheon, Republic of Korea) and treated with TGF-β1 (5 ng/mL). Subsequently, cells were co-incubated with or without PDE4 inhibitors for 48 h, followed by fixation with 4% paraformaldehyde for 10 min. After permeabilization with 0.1% Triton X-100 in 1× PBS and blocking with 5% bovine serum albumin, cells were incubated overnight with primary antibodies at 4 °C. The primary antibodies for immunocytochemistry were the same as those detailed in the Western blotting section, with dilutions adjusted accordingly: Procol1A1 (1:50), FN (1:500), α-SMA (1:500), MRTF-A (1:100), SRF (1:100).

Following three PBS washes (10 min each), cells were incubated with AlexaFluor488/594-conjugated secondary antibody (1:300 dilution, Molecular Probes, Eugene, OR, USA) at room temperature for 2 h. Cells were then mounted with Fluoroshield Mounting Medium containing DAPI (ab104139, Abcam) and visualized using a Zeiss LSM880 confocal laser scanning microscope. All experiments were conducted at least three times and representative images were shown. The fluorescence intensity ratio of Procol1A1, FN, α-SMA to DAPI was quantitatively analyzed using ImageJ software and the quantified results were presented as the mean intensity of five images from each group.

For quantification of the nuclear-to-cytoplasmic ratio, images were processed using ImageJ software. Cells were delineated using the Cell Mask stain, and the optical density of MRTF-A staining was measured, adjusted for cell area. The nuclear region, identified with DAPI stain, was similarly outlined for determining MRTF-A density within. The cytoplasmic fraction was calculated by subtracting the nuclear fraction from the total cell measurement. The nuclear-to-cytoplasmic ratio of MRTF-A was then determined by dividing the nuclear signal by the cytoplasmic signal. Analysis included average measurements in at least 10 cells per image in more than three independent experiments.

### 4.9. Statistical Analysis

Data in this study are presented as the mean ± standard error of the mean (SEM) based on a minimum of three independent experiments. For comparisons between two groups, the Mann–Whitney U test was utilized. In cases involving multiple comparisons, analysis of variance (ANOVA) was conducted, followed by Tukey’s post hoc test for specific group comparisons. Statistical significance was set at *p* values less than 0.05. All statistical analyses were performed using GraphPad Prism version 8.0 (GraphPad, San Diego, CA, USA).

## Data Availability

Data supporting the reported results are available upon reasonable request from the corresponding authors via e-mail.

## Supplementary Materials

The following supporting information can be downloaded at: https://www.mdpi.com/article/10.3390/ijms27167126/s1.

## Abbreviations

The following abbreviations are used in this manuscript:

TGF-β1	transforming growth factor-β1
PDE4	Phosphodiesterase 4
HIMFs	human intestinal myofibroblasts
FN	fibronectin
Procol1A1	procollagen type I alpha 1
SRF	serum response factor
MRTF-A	myocardin-related transcription factor A
α-SMA	α-smooth muscle actin
RhoA	Ras homolog family member A
ROCK	Rho-associated protein kinase
cAMP	cyclic adenosine monophosphate
PKA	protein kinase A
CREB	cAMP response element-binding protein
TBS-T	Tris-buffered saline with Tween
SEM	standard error of the mean
DSS	dextran sulfate sodium
EMT	epithelial–mesenchymal transition
EP2	prostaglandin E receptor
Epac	Exchange protein directly activated by cAMP
EP4	prostaglandin E receptor 4
ALK5 (TGFβR1)	Activin receptor-like kinase 5 (Transforming growth factor beta receptor 1)
ECM	extracellular matrix
MTT	3-(4,5-Dimethylthiazol-2-yl)-2,5-diphenyltetrazolium bromide
COPD	Chronic obstructive pulmonary disease
MMP	Matrix metalloproteinase
TNF-α	tumor necrosis factor alpha
CTGF	Connective tissue growth factor
CBP	CREB-binding protein
PGE2	prostaglandin E2
PDE	phosphodiesterase
