# Supplementary material for: Roflumilast Inhibits Fibrogenic Activation in Human Intestinal Myofibroblasts via Inhibition of Myocardin-Related Transcription Factor/Serum Response Factor Signaling
Source: Int J Mol Sci. 2026 Aug 8;27(16):7126. doi: 10.3390/ijms27167126 (PMC13513344; doi:10.3390/ijms27167126)
Supplement: Supplementary file 1 [file ijms-27-07126-s001.zip › ijms-4360777-supplementary methods and captions of figures.pdf]

Supplementary Methods

Identification the characteristics of HIMFs

For immunophenotypic characterization of isolated HIMFs, HIMFs (passage 5, 1.0×10^4 cells/well) were seeded onto the chamber slides (#30108, SPL life Sciences, Pocheon, Republic of Korea), and immunofluorescence staining was performed with antibodies: α-SMA (A2547, Sigma, Burlington, MA, USA), Vimentin (ab92547, Abcam, Cambridge, UK), Desmin (ab15200, Abcam). Nuclei were stained with DAPI and HIMFs were observed by Zeiss LSM880 confocal laser scanning microscope.

Quantification of PGE2 production

HIMFs were seeded at a density of 3×10^4 cells/well on a 24-well culture plate. The cells were treated with PDE4 inhibitor for 30 min and then exposed to 5 ng/ml TGF- β1 for 24 h. Medium was then collected, frozen, and stored at 80°C until assay. PGE2 production was measured by an Enzyme-linked immunosorbent assay (ELISA; R&D Systems, Minneapolis, MN, USA) by following the manufacturer's instructions. All assays were performed in triplicate, and data are shown as mean ± SEM.

Isolation of Human Intestinal Myofibroblast from patients with Crohn's disease and Culture

For validation experiments, primary intestinal myofibroblasts were additionally isolated from surgically resected intestinal tissues obtained from patients with Crohn's disease using the same protocol under IRB approval CHAMC 2020-07-02-1-017 (CHA Bundang Medical Center), with written informed consent. The tissues were obtained from inactive, non-fibrotic lesions as confirmed by gross and histopathological examination.

Co-culture of HIMF-CD with Roflumilast

The impact of roflumilast on TGF-β1-induced fibrosis in HIMF-CD was assessed by starving the cells in serum-free medium for 24 h, followed by 30 min pre-treatment with roflumilast and subsequent TGF-β1 (5 ng/mL) exposure for 24 h (RT-qPCR analysis) or 48 h (Western blot analysis).

Supplementary Figures captions

Figure S1. Original images of Western blots for Figure 2B. Red boxes indicate the cropped blots shown in Figure 2B. Membranes were cut to enable blotting for multiple antibodies. All samples were derived from the same experiment, and gels/blots were processed in parallel.

Figure S2. Original images of Western blots for Figure 5A. Red boxes indicate the cropped blots shown in Figure 5A. Membranes were cut to enable blotting for multiple antibodies. All samples were derived from the same experiment, and gels/blots were processed in parallel.

Figure S3. Original images of Western blots for Figure 7. Red boxes indicate the cropped blots shown in Figure 7A and B. Membranes were cut to enable blotting for multiple antibodies. All samples were derived from the same experiment, and gels/blots were processed in parallel.

Figure S4. Identification the characteristics of HIMFs. For immunophenotypic characterization of isolated HIMFs, HIMFs (passage 5) were stained with α-SMA, Vimentin, and Desmin antibodies and counterstained with DAPI. HIMFs were observed by Zeiss LSM880 confocal laser scanning microscope. HIMFs were identified as α-SMA and Vimentin positive and Desmin negative. Scale bars, 200 μm; original magnification, × 100.

Figure S5. Effects of PDE4 inhibitors on PGE2 release in HIMFs. HIMFs were treated with TGF-β1 (5 ng/mL) and co-cultured with or without 1 μM of rolipram, roflumilast, piclamilast or mesopram. Culture media were harvested after 24 h. PGE2 release is expressed in pg/ml. All values are means ± SEM for at least 3 independent experiments, each performed in triplicate. $^{\#}P < 0.05$ versus the control; $^{*}P < 0.05$ versus the TGF-β1 treatment only (ANOVA w/ Tukey).

Figure S6. TGF-β1-induced phosphorylation of Smad2 in time dependent. HIMFs were treated with TGF-β1 (5 ng/mL) for 30 min, 1 h, 2 h, 24 h and 48 h. (A) Representative western blots showing the protein expression of phosphorylated Smad2 (p-Smad2) and GAPDH as a loading control. (B) Quantitation of p-Smad2 from western blot analyses (n = 4). (C) Original images of Western blots in A. Red boxes indicate the cropped blots shown in Figure S6A. Membranes were cut to enable blotting for multiple antibodies. All samples were derived from the same experiment, and gels/blots were processed in parallel. Data are expressed as the mean ± SEM. $*P < 0.05$ versus each control (Mann–Whitney U test).

Figure S7. Roflumilast inhibit TGF-β1-induced fibrogenic activation of HIMF-CD. HIMF-CD were treated with TGF-β1 (5 ng/mL) and co-cultured with or without roflumilast (24 h for RT-qPCR analysis, 48 h for western blotting). (A) RT-qPCR analysis of the relative mRNA expression of collagen1A1 (*COL1A1*), FN (*FN1*), and α-smooth muscle actin (*ACTA2*). The data were normalized to *GAPDH* expression and expressed as relative values compared to the control (n = 4). (B) Representative western blots show the protein expression of procollagen1A1 (Procol1A1), fibronectin (FN), and α-smooth muscle actin (α-SMA), with GAPDH as a loading control. (C) Quantitation of Procol1A1, FN, and α-SMA from western blot analyses (n = 4). (D) Original images of Western blots in B. Red boxes indicate the cropped blots shown in Figure S7B. Membranes were cut to enable blotting for multiple antibodies. All samples were derived from the same experiment, and gels/blots were processed in parallel.

Data are expressed as the means ± SEM. $^{\#\#}P < 0.01$ and $^{\#\#\#}P < 0.001$ versus the control; $^{*}P < 0.05$, $^{**}P < 0.01$ and $^{***}P < 0.001$ versus the TGF-β1 treatment only (ANOVA w/ Tukey).
